# Brief intervention for obesity in primary care: how does student learning translate to the clinical context?

**DOI:** 10.15694/mep.2019.000016.1

**Published:** 2019-01-21

**Authors:** Kathleen Leedham-Green, Ann Wylie, Aikaterini Ageridou, Alec Knight, Emmanouil Smyrnakis

**Affiliations:** 1 King's College London; 2 Aristotle University of Thessaloniki

**Keywords:** obesity, behaviour change, social determinants of health, health coaching, overweight, health promotion, primary care, nutrition, undergraduate medical education, family medicine, brief intervention, sustainable healthcare, risk factors, non-communicable disease

## Abstract

This article was migrated. The article was marked as recommended.

There is an international call for more sustainable approaches to healthcare and for graduating doctors to develop the competencies to address the behavioural, psychological and social determinants of health. Obesity is a global challenge, and the case for preventative health is evident. There is growing evidence to support brief interventions for obesity in primary care. The feasibility and acceptability of teaching within classroom contexts have been demonstrated, however there are known barriers to adopting these approaches with patients. It is unclear how well classroom learning translates to the clinical context.

329 final year medical students from Aristotle University of Thessaloniki attended training that had been developed through action research processes at King’s College London and adapted to the local context. Students conducted brief interventions with 3,177 overweight or obese patients across 136 primary healthcare facilities over three rotations. Their reflective learning essays were coded for content and thematically analysed to illuminate their experiences.

Emergent themes include students’ insights into the drivers behind the obesity epidemic and psychosocial barriers to change; transformative experiences using patient-centred approaches to communication and behaviour change; progression in skills and attitudes to broaching obesity in clinical contexts; and insights into the factors that drive patient engagement. Their experiences indicate that facilitative approaches are acceptable to patients, and result in commitment to change where relevant to the patient’s agenda.

## Introduction

The prevalence of obesity in Greece is one of the highest in Europe, with 53.7% of the adult population being overweight or obese ( OECD, 2014) driven by complex risk factors ( Foresight report, 2007). In the same way that smoking cessation can be supported on both a population and an individual level ( Beattie, 1990), general practitioners are developing the skills to work collaboratively with their patients to co-create health ( Richards, Snow and Schroter, 2016) complementing wider public health programmes. It is imperative to work with patients to prevent disease ( Wylie and Holt, 2010), especially so in countries such as Greece where healthcare funding is limited ( OECD, 2016), thereby reducing the burden of disease and improving the sustainability of healthcare systems ( Mortimer, 2010).

There is growing evidence to support brief interventions for obesity in primary care ( Aveyard *et al.*, 2016). Chisholm et al. present a convincing case that obesity management is suboptimal in medical education ( A. Chisholm *et al.*, 2012b; A. Chisholm *et al.*, 2013). They argue that researching and evaluating practical applications of behaviour change theory lags advances in the theories themselves and have demonstrated that introducing evidentially informed health promotion education into medical curricula is both acceptible and feasible ( A. Chisholm *et al.*, 2016). Various studies show improvements after educational interventions in students’ confidence in their skills, their empathy towards people with obesity, and their knowledge of counselling strategies ( Poirier *et al.*, 2004; Kushner *et al.*, 2014). However, in these studies, students interact with standardised patients in role-play settings and there is little evidence about how this training translates into clinical practice. Assessment of learning according to the levels outlined by Kirkpatrick ( Kirkpatrick, 1975) tends to be low level, looking at self-reported confidence levels, or tests of knowledge, rather than assessing how learning has impacted on students’ behaviour in the clinical context or patient outcomes. There is a gap in the literature on how classroom learning with respect to obesity is applied in clinical contexts.

Research highlights barriers to broaching, suboptimal practices and attitudes amongst healthcare professionals who have not received health coaching training ( Gunther *et al.*, 2012; Nolan *et al.*, 2012; Blackburn *et al.*, 2015; Leiter *et al.*, 2015). These barriers include medical professionals and trainees avoiding behaviour change talk because of personal challenges, a lack of skills competency such as using directive rather than facilitative approaches, time prioritisation elsewhere, lack of knowledge of referral options and resources, delegation of responsibility (lack of role legitimacy), despondency due to previous negative experiences, and prioritising the doctor-patient relationship (fear of offence).

The cross-fertilisation of ideas between Implementation Science and Medical Education is an emergent and exciting concept ( Price *et al.*, 2015). Within Implementation Science there is an established framework for linking interventions to barriers and facilitators to evidence-based practice ( Michie, van Stralen and West, 2011). By using Michie’s framework, addressing capabilities, opportunities and motivations, and designing a theoretically and evidentially derived educational and clinical intervention, we have focused our educational programme so that it is more likely to be effective. This study evaluates this intervention through a thematic analysis of students’ reflective learning essays.

## Methods

### The intervention

**Figure 1.**
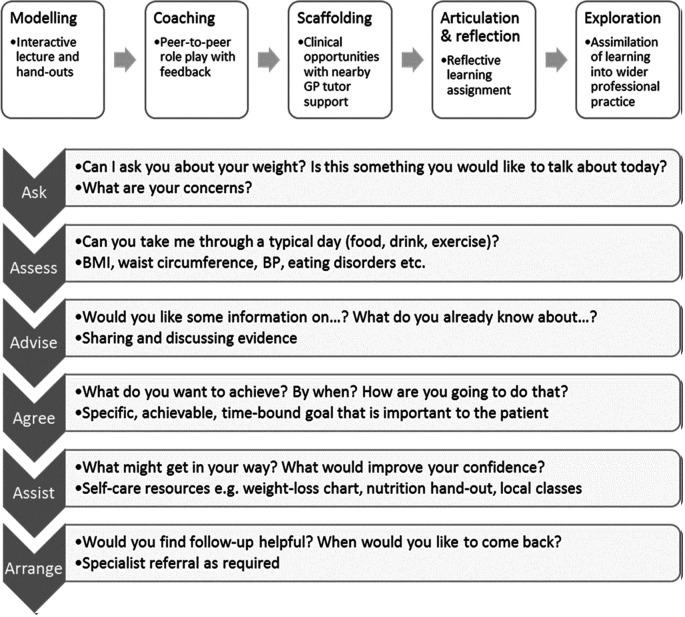
The educational and clinical intervention

This study was conducted at Aristotle University of Thessaloniki (AUTH) in collaboration with King’s College London (KCL). Experiences and processes from a curriculum implementation at KCL were shared with AUTH via webinar, e-learning and document exchange ( Leedham-Green, Pound and Wylie, 2016; Leedham-Green *et al.*, 2016). Teaching materials and methods, placement activities, and assessment processes were translated and adapted to the local context. The educational and clinical approaches are summarised in Figure 1. The educational approach follows the cognitive apprenticeship model ( Collins, Brown and Newman, 1989; Stalmeijer *et al.*, 2013).

Medical students in their final year (n= 329), on the first day of their 4-week General Practice (GP) placement, received training, through lecture and role-play, in motivational interviewing, behaviour-change theory, guidelines for nutrition and exercise, and a commonly used clinical framework for a brief intervention ( Glasgow, Emont and Miller, 2006). The framework was adapted to reflect current theory and guidelines to include eliciting a dietary and social history, offering tailored information rather than advising, patient-led goal setting and action-planning, as well as a facilitative approach to problem solving ( Resnicow and McMaster, 2012; Wolever *et al.*, 2013; NICE, 2014).

Students were divided into three ‘rotations’; October-November, February-March and May-June 2016. Students in each rotation were placed across 136 regional primary care facilities, and were tasked to discuss weight with patients who were overweight or obese (target 10 patients), completing a brief intervention where appropriate. Students were expected to record their patients’ BMI and waist circumference; past medical history; sociodemographic background; and a detailed dietary history. Consultation skills were facilitated by a skills sheet and goal-setting and action-planning proforma; dietary advice was facilitated by a patient handout, developed by dieticians at AUTH.

Students were set a portfolio assignment to reflect on their experiences, with prompts to discuss the main benefits and barriers they faced.

### Data sampling and analysis

Twenty students’ reflective essays from the first rotation were randomly selected, anonymized and translated into English through freely available online software, corrected by bilingual students. Content was coded using NVivo 11.0 software and saturation was determined when the rate at which new nodes were coded fell to less than 5% of the initial rate: this was achieved after 12 essays. This number of essays from each rotation was analysed in depth by two independent researchers, neither involved in the UK or Greek courses. All twenty essays from each cohort were investigated by two bilingual medical students from AUTH to verify language, coding and saturation. One additional theme was identified in this way. In total 36 essays were fully coded, with an additional 24 essays read and summarised as part of the checking process.

Coding was arranged into emergent themes and cross-checked through regular discussion with a third researcher from KCL. Research bias was mitigated through reflective discussions with a research mentor. Rotations were compared through a framework analysis that looked at the frequency of key nodes across rotations.

### Research ethics

The research had oversight of the Research Ethics Committee of the Aristotle University of Thessaloniki (approval number 227/28-3-2016).

## Results/Analysis

Content was grouped into four main categories of responses (themes) with sub-themes. Where appropriate we present our discussion in-line with the results, so that hypotheses can be seen alongside the relevant findings.

### Theme 1: Insights into obesogenic contexts

Students expressed insights into the complexity of patient contexts and how both personal and societal factors impact on eating and exercise behaviours.

#### Psychosocial factors

The economic climate in Greece was commonly attributed with having a negative impact on health, with students gaining insights into how unemployment and depression can result in inactivity and disordered eating behaviour. They saw that advice to join a gym or eat more fresh fish would be inappropriate to their patients in the current financial climate.

Students described how patients who were working long hours lacked the time or inclination to work out or cook from raw ingredients, instead consuming fast food that was calorie dense but low in nutrition.

#### Cultural erosion

There was concern about cultural challenges to healthy eating with popular media encouraging Westernisation of the traditional Mediterranean diet. Other impediments to implementing the advice in their nutritional handout included not knowing how to cook, and societal changes such as the tendency to live and eat alone.

#### Health literacy and aging

The age of patients was considered another factor, with students noticing that elderly patients tended to have lower health literacy, and to be less open to change, with many rural elderly having no education beyond primary school. Students found they needed more time to explain and clarify nutritional advice with these patients. Frailty was acknowledged as a barrier to exercise in this age group alongside a more fatalistic attitude to health.

### Theme 2: addressing the barriers to effective consultations

Students described a range of barriers and facilitators to effective consultations, including time, patient priorities, space, patient factors and their own skill as practitioners.

#### Time

In the first rotation, several students reported they did not have enough time to approach patients and ask them about their weight, because they were too busy with other obligations such as sitting in with clinics and learning to examine-diagnose-treat. By rotation three, no participants identified time as a barrier, perhaps reflecting greater acceptance of the task by their GP tutors.

#### Competing clinical priorities and tasks

Students saw that it was not always appropriate to approach patients if they were unwell and did not have the inclination or energy to devote to a discussion about their weight. Opportunistic discussions in the waiting room were often interrupted as patients were called for their appointment. The proforma questionnaire was described as an impediment, because it was long and the closed questions, which were often biomedical, impeded a free discussion. This has since been modified.

#### Broaching obesity with patients

Compared to experiences at KCL ( Leedham-Green, Pound and Wylie, 2016) where broaching obesity was seen as a major barrier to opportunistic health promotion, students appeared relatively comfortable raising the topic, barring privacy considerations. Their training on how to broach the subject was found to be supportive: ‘asking to ask’ rather than imposing the conversation, and focusing first on the patient’s concerns.

#### Patient factors

Students described patients who didn’t want to talk about their wider health problems or concerns; others who were willing to talk but were not interested in committing to change; and some who wanted to change but didn’t feel able or confident in doing so.

#### Physical space

Physical space and lack of privacy were important barriers, particularly in the first rotation, as students were approaching patients in the waiting area. Students stated that this sensitive conversation required privacy and trust, and the expectation to weigh and measure patients in a public space was not appropriate. By rotation three, this was no longer expressed as an issue and students described conversations taking place in other parts of the health centre, such as doctors’ offices or even outside in good weather. This barriers appears to have been addressed by local adjustments taking place.

#### Skills

This was the first time these medical students had used health coaching techniques. Some felt they were not sufficiently familiar with the approach and struggled to find the right words or to adopt the suggested techniques. Some students simply didn’t have the confidence to start conversations with patients. Students expressed insight into their need to develop their skills further and to get expert feedback suggesting on online forum, or observation by experts. As the three rotations progressed, lack of confidence in skills was less commonly expressed, perhaps reflecting cyclical improvements in pre-placement skills training and support from their GP tutors.

#### Student status

Some students reported difficulty engaging with patients which they attributed to their lack of professional authority or to their young age. One stated that their lack of status as a student meant that patients became ‘quarrelsome’. These concerns suggest that some students were still adopting a paternalistic stance, which relies on authority over their patient to impart advice, rather than a cooperative stance where information is shared and discussed.

#### Trust & rapport

Others felt their lack of prior relationship with patients made the conversation more difficult, showing insights into the need for trust and rapport. This was addressed in some instances through professional support by GP tutors who took the time to introduce patients to students. Others described building rapport themselves through a process of explanation and consent.

### Theme 3: Experiences with patients

Students reported a broad range of responses by patients. Many expressed surprise at how receptive patients were to having the opportunity to talk about their concerns.

#### Relevance to the patient’s agenda

It was acknowledged that patients were unlikely to engage if they were unwell and had come to see the GP about other matters. Students noted that younger patients engaged in more talk about change as they were more conscious of their weight, whereas some older patients appeared ‘set in their ways’. Patients who were already suffering from obesity-related pathology, such as diabetes, were described as particularly open to the conversation.

#### Focusing on the patient’s concerns

Students felt encouraged by patients who expressed ‘delight’ at the opportunity to talk about their concerns. Students reported patients as being ‘ *relieved to have the opportunity to discuss an issue that other people are embarrassed to raise with them*’, and to be ‘ *treated as a person rather than as a medical condition*’.

#### Student’s approach and acceptability to patients

Some students described more negative responses from patients than others, with some describing entirely positive experiences. It appears that the patient’s response was at least partly driven by the student’s approach, with attention to the patient’s agenda and rapport being associated with more positive responses.

#### Eliciting a commitment to change

Students expressed excitement at successful consultations where they felt they had helped mobilise and inform patients, noting ‘ *the satisfaction of helping your fellow man to address his health problem cannot be compared with any other emotion’.*

#### Willing to talk but not to change

Students acknowledged that despite positive engagement, not every consultation was likely to result in change, due to patients lacking either motivation or ability. They realised that some patients were unwilling to change their everyday habits, such as eating smaller meals or avoiding specific foods because of a social dimension to their food behaviour, such as eating with family and friends.

#### Moving from pre-contemplation to contemplation

Some recognised that even if a patient hadn’t committed to change, they had supported them in contemplating change and had shared helpful health information. Raising the topic also allowed family members to articulate their concerns, enabling conversations to take place that might not have otherwise.

### Theme 4: Student learning

This quote summarises our impression of student learning: *‘through this educational process we have been able to gain the insight that obesity is a multifaceted, complex condition with deep and unknown repercussions for the individual. Through our contact with patients we have learned new techniques of approach, new ways of thinking, and ways of involving them to act.*’

#### The relevance of trust and rapport

Throughout the essays, students highlighted the importance of a mutually trusting doctor-patient relationship as essential to the success of health-related discussions. By using this patient-centred model, which students described as ‘ *friendlier’* and ‘ *holistic’* they felt able to build trust and rapport. Students described incremental increases in confidence and in their ability to build rapport, based on their experiences using patient-centered approaches to communication.

Some stated that patients had a responsibility to trust their doctors and collaborate with them to achieve their goals. They felt patients had a duty to listen to evidence relating to the feasibility of their action plan, such as avoiding extreme diets.

#### Understanding the patient’s perspective and co-creating solutions

Students felt that by listening to their patients’ problems it helped them to understand their desires and needs, and thereby discuss meaningful goals. By establishing a two-way conversation, they felt able to offer tailored rather than generic advice. By starting the conversation with the patients’ concerns, students became aware of the psychosocial difficulties that patients faced. Students expressed frustration at these difficulties rather than judgement. Their language indicated a collaborative, participatory stance: ‘ *Working with the patient helped us to exchange information, to voice his concerns and, most importantly, to collaborate towards finding satisfactory solutions.*’

#### Transformative experiences

Some students described transformative learning experiences ( Mezirow, 1990) using language such as ‘ *new way of thinking’.* The change in emphasis from pathology-based to health-based practice was also transformational for some: *‘.I improved my communication skills with patients and experienced their real desires, fears, anxieties and expectations, something that hardly ever happens in the hospital environment, ultimately gaining a profound insight’.*

Students expressed insights into sectors of society that they hadn’t previously engaged with *‘I understood that the doctor should be aware of the difference, the value of diversity and the satisfaction derived from learning from people from different areas of society’.*

#### Advanced communication skills

Students valued strategies associated with motivational interviewing and felt that the communication techniques they had learnt would be widely applicable in their future practice to support patient engagement in health: ‘ *Each day, whether or not I am conversing with patients who are overweight, I now try to adapt my way of communication, my choice of words, and my body language, depending on the patient I am facing.’*

After this intervention, students felt they were more comfortable broaching sensitive topics in general without causing embarrassment or upset to their patients *.*

#### Developing professional identities

Students described changes to their professional identity, reformulating their concepts of the family doctor as: ‘ *safeguarding the health of the local community*’; ‘ *influencing patients’ lives for the better*’; and ‘ *as a specialist in information and counselling of patients’.*

A minority described obesity-related health promotion as the role of other members of the healthcare team, and outside their future professional role.

Concepts of preventative care

Students felt that behaviour change as a skill was so important that it ‘ *should be present in every clinician and used systematically’.* Addressing obesity was seen as vitally important to the future of healthcare. Students wrote about the determinants of health and recognised that *‘medication alone is not enough to effectively tackle widespread diseases’.*

Students expressed insights into how change can be further supported through self-care and follow-up. Some students shared their goal-setting sheets with patients while others used them to support follow-up within the patient’s records at practice.

Students mainly embraced the intervention which they saw as useful not only in primary prevention, but also in the management of obesity-related morbidities such as arthritis, diabetes and cardiovascular disease.

One student who described his/her self as overweight, was opposed to the preventative model, because they felt that it was ‘impolite’ to broach the topic proactively, preferring to raise it in the context of dyslipidaemia or diabetes.

#### Other themes of learning

Other themes of learning included: the value of exchanging information rather than advising, eliciting concerns, working towards collaborative solution that involved the patient, implementing protocols and care pathways, public health, community medicine, and how to translate theoretical knowledge into clinical care.

## Discussion

This intervention has addressed many of the known barriers to consulting about obesity in primary care ( Anna Chisholm *et al.*, 2012a; Blackburn *et al.*, 2015) which include difficulty broaching, fear of offence, lack of evidence-based health coaching skills, lack of knowledge as to how to treat obesity, and a tendency to only address obesity in response to pathology rather than proactively. The intervention also substantially addresses lack of time and resources, as medical students have been mobilised to support primary care clinicians as part of their education. The barrier of role legitimacy appeared to reduce as students normalised this activity, encouraged by positive patient responses, and support from their host GP practices. Personal challenges, such as being overweight, and interpersonal dynamics, including a tendency amongst some students for paternalistic advice, warrant further attention.

Gunther et al. ( Gunther *et al.*, 2012) interviewed patients as well as healthcare professionals and identified an additional barrier to consulting about obesity: trust and weight bias. Bias was not a strong theme in our findings or in papers that concentrate solely on the views of healthcare professionals ( Anna Chisholm *et al.*, 2012a; Blackburn *et al.*, 2015). This raises the interesting questions of whether weight stigma is felt or enacted ( Goffman, 1963; Scambler, 2004) and whether weight bias amongst medical professionals is implicit or explicit ( Phelan *et al.*, 2014). Our intervention may have addressed students’ weight bias to some extent, consistent with findings that weight bias reduces with favourable contact experience with obese individuals ( Meadows *et al.*, 2017).

Follow-up and ongoing social support for change, which are important aspects of evidence-based practice, were missing from this intervention. This was not helped by many rural practices having paper records, or no patient records, with no facility for automated patient recall and limited access to social-prescribing resources. An investment in primary care infrastructure is required. Individual GP practices may need to develop novel solutions in the short-term.

Importantly, our analysis indicates that patient engagement is driven at least partly by the approach of the student. Broaching conversations about obesity proactively appears to be acceptable to patients where trust and rapport have been built, where there is sensitivity to the patient’s agenda, and where patient-centred approaches are used.

### Strengths and limitations

Presenting our results as a thematic analysis has been helpful in systematically drawing out the full range of students’ experiences. It is challenging however to convey any sense of narrative or the relative weighting of different experiences. A balanced argument within a single essay might be lost, or the negative experience of one student being given the same attention as the positive experiences of a much larger number of students. This has been mitigated as far as possible in our discussion, where we try to bring out our overarching impression of this data.

Another limitation is the data source: self-reported experiences of clinical encounters. Students may have presented their experiences in a positive light, as this was a course assignment, however its formative nature will have enabled students to write relatively freely and critically.

A strength of this study is its scale, enabling researchers to be confident about saturation of themes. Translation challenges have been greatly facilitated by a bilingual researcher with support from two bilingual medical students.

Further research is required to explore the patient’s perspective in a more direct way, and to evaluate patient outcomes at follow up.

## Conclusion

This intervention has supported medical students in normalising conversations about lifestyle in the clinical consultation, and in developing facilitative, patient-centred approaches to clinical communication. For patients and their families, this has enabled discussions about the role of weight management in health, and engagement in conversations inviting change that might not have otherwise happened. The barrier of broaching the subject of obesity in a way that is acceptable to patients appears to be largely overcome, through offering rather than imposing the conversation and focusing on the patient’s concerns. Previous research has demonstrated how health coaching techniques can be successfully taught to medical students through classroom-based activities and role play, however, we have demonstrated how students are able to apply, develop and hone these skills with patients in clinical contexts. By requiring students to submit reflective essays, students gained the opportunity to articulate and reflect on their experiences and to share their learning with their GP teachers, creating opportunities for ‘trickle-up’ learning to teaching practices. Faculty also benefited from having access to their reflections, supporting curricular evaluation and improvement.

This approach has been successful in addressing the known barriers to consulting about obesity in primary care in two international contexts. It appears effective, feasible and transferable.

## Take Home Messages

• Broaching obesity in clinical contexts appears acceptable to patients where rapport and trust have been established; the patient’s privacy and autonomy are respected; and the primary focus is on the patient’s concerns.
• Students can assimilate patient-centred health coaching skills through a cognitive apprenticeship model.
• Students value health coaching skills which they consider to be helpful to patients and widely applicable to their future clinical practice.
• Issues with this new curricular element had largely resolved within three rotations and it appears acceptable and sustainable in its modified form.
• Reflective learning essays can be helpful in disseminating learning to networked GP practices.

## Notes On Contributors

Dr Kathleen E Leedham-Green MBBS MA Clin Ed is a Medical Education Research Fellow at King’s College London and Imperial College London. Her publications and research interests include sustainable healthcare, health promotion, patient agency, quality improvement and healthcare equity. She has won two international education prizes for her work on obesity. https://orcid.org/0000-0002-5010-3257

Aikaterini Ageridou is an Associate Registered Nutritionist and she is currently doing her Masters in dietetics at King’s College London. She is bilingual English-Greek and her contribution was funded as part of the King’s Undergraduate Research Fellowship scheme.

Dr Alec Knight PhD is a Teaching Fellow in Public Health in the Faculty of Life Sciences & Medicine, King’s College London. Alec has active research interests in implementation and improvement science, medical education, and work and organisational psychology applied to healthcare, and has published numerous refereed papers across these fields. https://orcid.org/0000-0002-2937-436X

Dr Ann Wylie PhD is a medical educationalist, focused on Primary Care, Public Health, Health Promotion and Global Health, with a special interest in lifestyle issues, the obesity epidemic and social determinants of health as integral to medical education. https://orcid.org/0000-0001-6626-0751

Dr Emmanouil Smyrnakis PhD, MSc, is an Assistant Professor in Primary Healthcare and Medical Education at Aristotle University of Thessaloniki Medical School, where he leads the Primary Healthcare teaching and research network. He has published extensively on health promotion, disease prevention and the socioeconomic determinants of health in Greece. https://orcid.org/0000-0002-9772-4595

## Appendices

### Online Resource

Online educational resource from King’s Health Partners outlining the patient-centred approach to addressing obesity in a clinical consultation.

#### Glossary Terms

*Rotation*: in the traditional block clerkship model of medical education, students are expected to rotate between the major disciplines of medicine which are constructed in blocks, each relating to a clinical placement. Each block is constructed in set units of time so that students rotate between blocks at the same time. A stage is completed when all students have rotated between all clinical blocks.

*Cognitive apprenticeship model*: This model makes explicit the tacit educational processes that occur during the journey from novice to expert: modelling, coaching, scaffolding, articulation, reflection and exploration https://www.learning-theories.com/cognitive-apprenticeship-collins-et-al.html
