# Supplementary material for: Brief intervention for obesity in primary care: how does student learning translate to the clinical context?
Source: MedEdPublish (2016). 2019 Jan 21;8:16. [Version 1] doi: 10.15694/mep.2019.000016.1 (PMC10712503; doi:10.15694/mep.2019.000016.1)
Supplement: Peer review history

# Reviewer report: Reviewer response for version 1

**DOI:** 10.21956/mep.19760.r29665

**Published:** 2019-06-15

**Authors:** Ken Masters

**Affiliations:** 1 Sultan Qaboos University

This review has been migrated. The reviewer awarded 3 stars out of 5 An interesting paper that evaluates the extent to which a brief intervention for obesity in primary care translates into the clinical context. The authors begin by highlighting the particular problem of obesity in Greece (where the study is set), and the literature that currently indicates that short interventions can have an impact, but these are often studies involving role-play with standardised patients. This study evaluates the process through student self-reflective essays based on clinical experiences.While the study is useful, and the results are interesting, there are a few issues with the paper:• The authors state that “saturation was determined when the rate at which new nodes were coded fell to less than 5% of the initial rate: this was achieved after 12 essays,” but do not cite a reference for this method of determining saturation. I have not seen this method of saturation determination (using a pre-set percentage of 5%) elsewhere, and I suspect it is quite unusual. How was 5% determined to be the optimal level, and what is the “initial rate” that is described here? Does this mean that if the essay added fewer than 5% new nodes to the first essay analysed, then saturation point will be assumed to have been reached? Given that setting a percentage of 5% (or any percentage, apart from 0%) is so unusual, I would like the authors to explain the process in a little more detail, and also cite a reference to this approach, not merely another study that may use it, but a study that explains the theoretical rationale and/or validates it.• Related to this, it appears that, for each cohort, saturation point was reached at exactly the same number of essays (12) each time. Or did the analysis of the first cohort determine that 12 was to be used, and so the other cohorts were also limited to 12, irrespective of whether or not saturation point had been reached?• While the presentation of the themes is useful, qualitative data are best presented with examples from the raw data. So, it would have been better if the authors had supplied one or two examples from the data for each theme. This is done to some extent in the later part of the Results, but not nearly enough. (If the authors do not wish to break the flow of the narrative, then these could be included in an appendix).• The interpretation that ‘quarrelsome’ patients “suggest that some students were still adopting a paternalistic stance” can only really be validated by a deeper exploration of the raw data, which, unfortunately, the authors have not provided. Patients, like any people, can become quarrelsome for a range of reasons.So, overall, an interesting study, but the authors do themselves a disservice by using an unusual (and not well-explained or justified) method of determining data saturation, and also by a lack of examples in their data presentation.

Reviewer Expertise:

NA

No decision status is available

# Reviewer report: Reviewer response for version 1

**DOI:** 10.21956/mep.19760.r29664

**Published:** 2019-03-03

**Authors:** Arti Maini

**Affiliations:** 1 Imperial College London

This review has been migrated. The reviewer awarded 5 stars out of 5 This is a very interesting and timely paper. The topic of health coaching for behaviour change is very relevant in the healthcare landscape currently and therefore in medical education. The abstract provides a readable and concise description of the paper, include relevant key messages. The introduction provides a helpful background, referencing relevant literature, and highlights clearly the goals of the paper. The methods are described in detail. The presentation of results was logical and systematic, and it was helpful to see the commentary for each theme identified. The discussion brings together insights from the results, exploring these more deeply, and the conclusion is aligned to the results and discussions sections. Strengths and weaknesses of the study have been identified. I believe this paper makes an important contribution to the field of health coaching and its application in healthcare settings, highlighting implications for undergraduate medical training.

Reviewer Expertise:

NA

No decision status is available

# Reviewer report: Reviewer response for version 1

**DOI:** 10.21956/mep.19760.r29663

**Published:** 2019-01-22

**Authors:** Trevor Gibbs

**Affiliations:** 1 AMEE

This review has been migrated. The reviewer awarded 4 stars out of 5 A very interesting and well written and well researched paper covering an important , yet difficult subject. It was good to see the trans-national approach to research and the possibility of transformative research. I learned a lot from this paper and felt that their use of theory was very helpful. The authors clearly expressed the positive factors that came out of this paper as well as clearly outlining the difficulties and possible weaknesses. I would imagine that anyone reading this paper would finally have to say- "what about the patient?" , so it was good to see the possibility that the authors would consider developing this project to evaluate the research to at least Kirkpatrick's levels 3 and 4.

Reviewer Expertise:

NA

No decision status is available
